# The Development of Keratopathy after Surgery-Indicated Chronic Rhinosinusitis: A Population-Based Cohort Study

**DOI:** 10.3390/ijerph16071218

**Published:** 2019-04-05

**Authors:** Hung-Jui Hsu, Chia-Yi Lee, Kun-Lin Yang, Hung-Chi Chen, Chi-Chin Sun, Jing-Yang Huang, Hung-Yu Lin, Shun-Fa Yang

**Affiliations:** 1 Institute of Medicine, Chung Shan Medical University, Taichung 402, Taiwan; juihee@hotmail.com (H.-J.H.); anthonyhungyulin@hotmail.com (H.-Y.L.); 2 Department of Ophthalmology, Show Chwan Memorial Hospital, Changhua 500, Taiwan; ao6u.3msn@hotmail.com; 3 Department of Optometry, College of Medicine and Life Science, Chung Hwa University of Medical Technology, Tainan 717, Taiwan; 4 Department of Otolaryngology–Head and Neck Surgery, Kaohsiung Chang Gung Memorial Hospital, Kaohsiung 833, Taiwan; mp9371@cgmh.org.tw; 5 Department of Ophthalmology, Chang Gung Memorial Hospital, Linkou 333, Taiwan; mr3756@cgmh.org.tw; 6 Department of Medicine, Chang Gung University College of Medicine, Taoyuan 333, Taiwan; 7 Center for Tissue Engineering, Chang Gung Memorial Hospital, Linkou 333, Taiwan; 8 Department of Ophthalmology, Chang Gung Memorial Hospital, Keelung 204, Taiwan; arvin.sun@msa.hinet.net; 9 Department of Chinese Medicine, Chang Gung University, Taoyuan 333, Taiwan; 10 Department of Medical Research, Chung Shan Medical University Hospital, Taichung 402, Taiwan; wchinyang@gmail.com; 11 Department of Optometry, Chung Shan Medical University, Taichung 402, Taiwan; 12 Department of Exercise and Health Promotion, Chung Chou University of Science and Technology, Changhua 500, Taiwan

**Keywords:** chronic rhinosinusitis, functional endoscopic sinus surgery, keratopathy, cornea, population-based

## Abstract

The aim of the present study was to evaluate the risk of developing keratopathy in patients with surgery-indicated chronic rhinosinusitis (CRS) via the National Health Insurance Research Database in Taiwan. Patients with a diagnostic code of CRS and who received functional endoscopic sinus surgery (FESS) were considered to have surgery-indicated CRS. The exclusion criteria were legal blindness, an ocular tumor, eyeball removal or previous keratopathy, and each individual in the study group was matched to four non-CRS patients by age and sex. The outcome was set as the occurrence of keratopathy according to the diagnostic codes after the index date. Cox proportional hazard regression was used for statistical analysis. A total of 6053 patients with surgery-indicated CRS and another 24,212 non-CRS individuals were enrolled after exclusions. The age and sex distributions were identical between the two groups due to matching, while comorbidities, including hypertension, diabetes mellitus, and other cardiovascular disorders, were significantly higher in the study group. There were 231 episodes of keratopathy in the study group, and 695 episodes of keratopathy in the control group after the index date, for which study group showed a significantly higher rate of developing keratopathy with an adjusted hazard ratio of 1.208 and a higher cumulative probability. In subgroup analysis, female sex with surgery-indicated CRS showed a significantly greater risk of developing keratopathy. In conclusion, surgery-indicated CRS that needs FESS to relieve symptoms is a potential risk factor for keratopathy.

## 1. Introduction

Chronic rhinosinusitis (CRS) is an inflammatory disease in the paranasal sinuses that persists for at least 12 weeks [1] and is found in more than 10 percent of the population [2]. The clinical presentation of CRS includes nasal discharge, pain in the facial region, reduction in the smell, headache, and shortness of breath [1,3]. Cranial nerve disorders, such as the alteration of trigeminal function, may occur in patients with CRS [4,5]. In its severe form, the infection and inflammation of the paranasal sinus in CRS may lead to a dreaded intracranial infection [6].

Both medical and surgical management have been utilized to treat CRS [7]. Topical corticosteroid therapy, oral corticosteroid administration, and antibiotic treatment with macrolide have been applied to treat CRS with acceptable outcomes [3,7]. Functional endoscopic sinus surgery (FESS) is a well-established intervention for CRS that is severe and refractory to medical management [8,9,10]. Nevertheless, the recovery of nasal sinus mucosa in patients with CRS is incomplete one year after FESS management [3]. Moreover, patients with some risk factors, such as higher Lund-Mackay computed tompgraphy scores and fungal-induced CRS, may still experience poor quality of life or persistent nasal polyp formation even after successful FESS intervention [11,12]. The above evidence suggests that the effect of severe CRS could persist despite FESS.

For ocular complications in CRS, both preseptal and visual-threatening orbital cellulitis were found in previous research [13]. Besides, dacryocystitis has also been reported after the development of CRS [14]. For the external eye region, however, only one case with concurrent keratoconjunctivitis and CRS was found previously [15]. Because severe CRS can influence adjacent structures, it is possible that severe CRS may result in certain corneal and conjunctival lesions.

The aim of the current study was to evaluate the development of keratopathy after surgery-indicated CRS that received FESS via the National Health Insurance Research Database (NHIRD) in Taiwan. The effect of CRS disease interval and other potential risk factors for developing keratopathy were also investigated.

## 2. Materials and Methods

### 2.1. Data Source

This retrospective, population-based cohort study was approved by the National Health Insurance Administration and the Institutional Review Board of Chung Shan Medical University. Provided by the Taiwan National Health Research Institutes, the NHIRD contains data on insurance claims from more than 99% of Taiwan’s population. The claims data used in the current study were obtained from the 2016 version of the Longitudinal Health Insurance Database (LHID 2016). The LHID 2016 contains data on two million patients randomly sampled from the NHIRD registry since 2005. The LHID 2016 data were linked from 1 January 2000 to 31 December 2016, and both the International Classification of Diseases, Ninth Revision (ICD-9) and International Classification of Diseases, Tenth Revision (ICD-10) were used for disease diagnosis. Details on the medications prescribed to the patients and patient demographics, socioeconomic status, and residence are also available in the NHIRD.

### 2.2. Patient Selection

Patients were defined as having surgery-indicated CRS if their medical records indicated (1) a history of CRS (ICD-9 codes: 473.x, ICD-10 codes: J32.x), (2) arrangement for FESS (procedure codes: 65013B, 65015B, 65063B, 65064B) within two years of diagnosis of CRS, (3) the usage of corticosteroids or antibiotics for at least two years from the diagnosis of CRS, and (4) receipt of the CRS diagnosis by an otorhinolaryngologist (department code: 09). The index date was set as the date two years after the diagnosis of CRS. To more accurately elucidate the association between surgery-indicated CRS and keratopathy, the following exclusion criteria were applied: (1) a diagnosis of legal blindness (ICD-9 codes: 369.4, ICD-10 codes: H54.0x, H54.1x, H54.4x, H54.8) at any time, (2) a diagnosis of ocular tumors (ICD-9 codes: 190.0–190.9, ICD-10 codes: C69.x) at any time, (3) a diagnosis of severe ocular trauma (ICD-9 codes: 871.0–871.2, 871.4–871.9, ICD-10 codes: S05.2x–S05.6x) at any time, (4) any type of eyeball removal surgery or diagnosis of anophthalmos (ICD-9 codes: 16.3x, 16.4x, 16.5x, 871.3, ICD-10 codes: Q11.1, S05.7x, Z90.01 plus procedure codes: 85001C, 85002C, 86808B) before the index date, or (5) a diagnosis of any type of keratopathy (ICD-9 codes: 370.x, 371.x, ICD-10: H16.x, H17.x, H18.x) before the index date. In addition, each individual in the study group was age- and sex-matched with four non-CRS individuals, as discussed in the following sections, which constituted the control group. Patients with surgery-indicated CRS who could not be matched with four non-CRS patients were excluded.

### 2.3. Main Outcome Measurement

The development of keratopathy is the main outcome in the current study and was based on the keratopathy-related diagnostic codes (ICD-9 codes: 370.0x, 370.2x, 370.3x, 370.4x, 370.5x, 370.6x, 370.8, 370.9, 371.0x, 371.21-371.23, ICD-10 codes: H16.0x H16.1x, H16.20x, H16.23x–H16.29x, H16.3x–H16.9, H17.1x, H17.8x, H17.9, H18.1x, H18.22x, H18.23x). In practice, ICD-9 codes for “unspecific corneal disorder (ICD-9 codes: 371.9, ICD-10 codes: H18.9)” may also be considered for some forms of keratopathy, but these codes were eliminated to prevent overestimation and confusion. Furthermore, only patients who received the abovementioned diagnostic codes by an ophthalmologist (department code: 10) were considered to have the outcome and included in the study.

### 2.4. Demographic Variables and Co-Morbidities

To standardize the health condition of participants, we also considered the effects of demographic conditions (i.e., age, sex, and income level) and the following systemic co-morbidities, according to our Modified Deyo–Charlson co-morbidity index in the multivariate analysis model: hypertension (ICD-9 codes: 401.x–405.x, ICD-10 codes: I10, I11.x, I13.x, I15.x, I16.x, I87.3x, I97.3x, O10.x, O11.x, O13.x, O16.x), diabetes mellitus (ICD-9 codes: 250.x, 277.7, ICD-10 codes: O24.4, E11.x, E13.x, E88.81), ischemic heart diseases (ICD-9 codes: 410.x, 412.x, 414.0, 414.0x, 414.2, 414.3, 414.4, 414.8, 414.9, ICD-10 codes: I20.x–I25.x), hyperlipidemia (ICD-9 codes: 272.0, 272.1, 272.2, 272.4, 272.9, ICD-10 codes: E78.0x, E78.1, E78.2, E78.3, E78.4x, E78.5, E78.70, E78.79, E78.89, E78.9), congestive heart failure (ICD-9 codes: 398.91, 402.01, 402.11, 402.91, 404.01, 404.03, 404.11, 404.13, 404.91, 404.93, 425.4–425.9, 428.x, ICD-10 codes: I50.2x, I50.3x, I50.4x, I50.84, I50.89, I50.9), peripheral vascular disease (ICD-9 codes: 093.0, 437.3, 440.x, 441.x, 443.1–443.9, 47.1, 557.1, 557.9, V43.4, ICD-10 codes: E75.21, I73.x, I79.8, T82.856, Z95.82x, Z98.62), cerebrovascular disease (ICD-9 codes: 362.34, 430.x–438.x, ICD-10 codes: G46.x, I60.x-I66.x, I67.0, I67.1, I67.2, I67.6, I67.81, I67.82, I67.84x, I67.89, I67.9), dementia (ICD-9 codes 290.x, 294.1, 331.2, ICD-10 codes: F01.x, F02.x, F03.x, G31.x), chronic pulmonary disease, including asthma (ICD-9 codes: 416.8, 416.9, 490.x–505.x, 506.4, 508.1, 508.8, ICD-10 codes: J41.x, J42, J43.x, J44.x, J47.x), rheumatic disease (ICD-9 codes: 446.5, 710.0, 710.1, 710.3, 710.4, 714.0–714.2, 714.8, 725.x, ICD-10 codes: M05.1x, M05.2x-M05.9, M31.6, M32.1x, M32.8, M32.9, M33.03, M33.13, M33.2x, M33.90, M33.93, M34.0x, M34.1x, M34.9, M35.3), and peptic ulcer disease (ICD-9 codes: 531.x–534.x, ICD-10 codes: K25.x-K28.x). To further standardize the ocular condition, dry eye disease (DED) (ICD-9 codes: 370.33, 370.34, 372.53, 375.15, 710.2, ICD-10 codes: H04.12x, H11.14x, H16.21x, H16.22x, M35.00, M35.01), uveitis (ICD-9 codes: 360.12, 363.0x, 363.1x, 363.20, 363.21, 363.22, 364.0x, 364.1x, 364.2x, 364.3, ICD-10 codes: H20.x, H30.0x, H30.1x, H30.2x, H30.8x, H30.9x, H44.11x), and glaucoma (ICD-9 codes: 365.1x, 365.2x, 365.7x, 365.9, ICD-10 codes: H40.1x, H40.2x, H40.89, H40.9) were also considered in the multivariate model. We longitudinally traced the data from the index date until the date of keratopathy diagnosis, withdrawal from the National Health Insurance program, or 31 December 2016.

### 2.5. Statistical Analysis

SAS version 9.4 (SAS Institute Inc, Cary, NC, USA) was employed for all analyses. After age and sex-matching at 1:4 ratio of the study and control groups, the incidence rate, crude relative risk, and corresponding 95% confidence intervals (CI) were calculated using Poisson regression. In the next step, Cox proportional hazard regression was adopted to compute adjusted hazard ratios (aHR) by incorporating the aforementioned demographic data, prominent ocular diseases, and systemic comorbidities in the multivariate model. Moreover, the sensitivity analysis with aHR which stratified by the sex and age subgroups was also conducted for the subgroup analysis. We plotted Kaplan–Meier curves to indicate the cumulative incidence proportion of keratopathy between the study and control groups, and used the log rank test to determine the significant difference between the survival curves. Because most patients in the NHIRD are Han Taiwanese, the race was not considered a covariate. Statistical significance was set at *p* < 0.05.

## 3. Results

**Figure 1:**
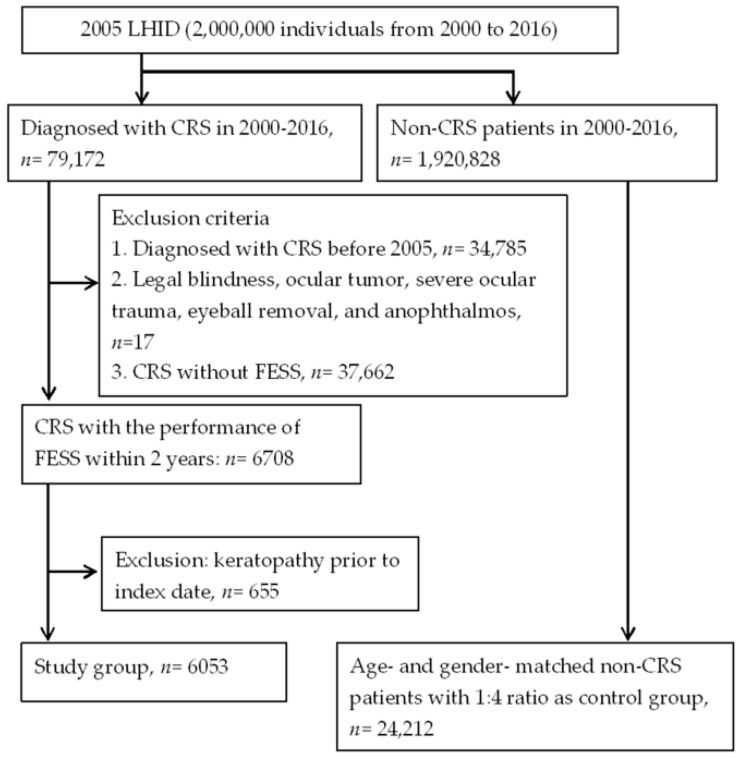
The flowchart of patient selection. LHID: Longitudinal Health Insurance Database; CRS: chronic rhinosinusitis; FESS: functional endoscopic sinus surgery.

A total of 6053 patients with surgery-indicated CRS were enrolled in the study group after exclusion, and other 24,212 individuals were enrolled in the control group. The flowchart of patient selection is shown in Figure 1. The age and sex ratios were identical due to the matching process, and the different comorbidities in the study and control group are shown in Table 1.

**Figure 2:**
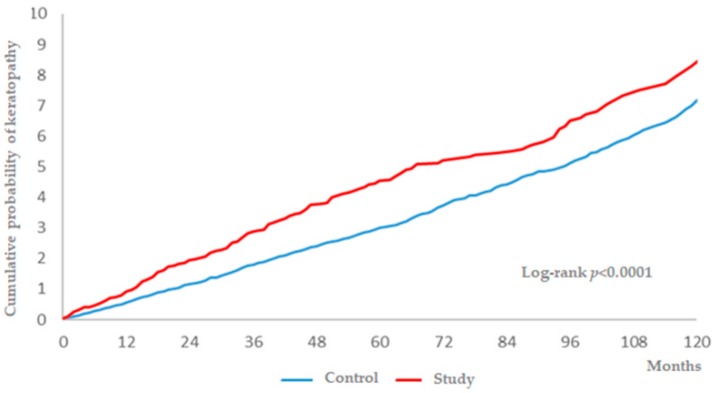
The cumulative probability of keratopathy between the study and control groups.

There were 231 and 695 events of keratopathy in the study group and the control group, while the study group had higher incidence rate (74.35) and crude relative risk (1.345) (Table 2). Moreover, the study group showed a significant aHR (1.208, 95% CI: 1.038–1.406) compared to the control group after adjusting for demographic data, disease interval, prominent ocular diseases, and systemic comorbidities (Table 3), and a significantly higher cumulative probability was also revealed in the study group (*p* < 0.0001) (Figure 2). In addition to surgery-indicated CRS, hypertension, chronic pulmonary diseases, peptic ulcer disease, and DED were also prominently related to the development of keratopathy (Table 3). In the subgroup analysis, females with surgery-indicated CRS had a significantly higher aHR (1.287, 95% CI: 1.038–1.595) for developing keratopathy, while only a marginally significant value was found for males with surgery-indicated CRS (Table 4).

## 4. Discussion

Briefly, CRS that requires surgical management to relieve symptoms significantly increased the risk of developing keratopathy in the current study after adjusting for multiple potential risk factors. Moreover, the chance of developing keratopathy was elevated in several diseases, including cardiovascular disorders, pulmonary diseases, and DED in the multivariate model.

Several mechanisms may lead to keratopathy. At first, inflammatory status is an important part of the pathophysiology of keratopathy, in which the elevated inflammatory mediators can be found in both the auto-immune and infectious keratopathies [16,17]. Similarly, the CRS has been regarded as an inflammatory disease recently, and both the inflammatory-related epithelial barrier dysfunction and immunoglobulin E production were proposed as the etiologic mechanisms for CRS [18]. Moreover, same inflammatory mediators, such as interleukin and tumor necrosis factor-alpha, are shown to be elevated in CRS and keratopathy in preceding researches [17,18,19]. Accordingly, the elevated inflammatory process in CRS might also raise the possibility of corneal damage. In addition, the infections accounting for certain types of keratopathies [20,21] and lesions like liver abscesses and orbital infections could lead to keratitis via hematogenous route [22,23]. Although accounting for much fewer cases compared to inflammatory disorders, infection is still one of the etiologies of CRS [24]. Thus, it is possible that the microorganisms that spread from infectious CRS to the cornea via either the anastomosis of an anterior ethmoidal artery-posterior ethmoidal artery-sphenopalatine artery or cavernous sinus-ophthalmic vein route then lead to keratopathy. On the other hand, some autoimmune diseases, such as Wegener granulomatosis, lead to both corneal lesions and CRS [25], and CRS might indicate an underlying autoimmune disease that may lead to keratopathy. As a consequence, the presence of CRS, especially severe cases that need surgery to manage, may lead to keratopathy, which was supported by the current study.

For the relationship between CRS and keratopathy, only concurrent keratoconjunctivitis and CRS had been found in previous research [15]. In the current study, surgery-indicated CRS that required FESS management showed an increased risk of developing keratopathy with a significantly higher aHR after controlling the effect of several potential risk factors in the multivariate analysis. To our knowledge, this study is a preliminary investigation that demonstrated a correlation between surgery-indicated CRS and keratopathy. In addition, only those episodes of keratopathy that occurred after arranging for FESS were included, and the increase in the risk of developing keratopathy with longer disease period of CRS was also evaluated which presented with the higher cumulative probability in the study group. Thus, a causal relationship is supported. For the difference in correlation between the two diseases in the different sexes, although the aHR in males was non-significant, the aHR was similar to that in the female population with a similar interaction *p*-value, and the lower limit also showed marginal significance. We speculated that surgery-indicated CRS elevated the risk of developing keratopathy in males with a longer follow-up interval.

Several disorders were also related to the development of keratopathy according to the multivariate analysis in the current study. DED revealed significantly higher aHR for the occurrence of keratopathy in the current study. Because DED will lead to inflammation and damage of the ocular surface [26], the relationship between DED and keratopathy in the current study is reasonable and has been illustrated in a previous study [27]. Concerning the influence of glaucoma on the cornea, an acute attack of glaucoma may lead to corneal decompensation in patients with an impaired corneal endothelium [28]. Certain anti-glaucomatous agents can also contribute to damage the ocular surface and keratoepitheliopathy, such as superficial punctate keratitis [29]. In the current study, the aHR of glaucoma was also elevated, although not significantly. On the other hand, uveitis also did not show a significant effect on the occurrence of keratopathy. This phenomenon might result from the exclusion of patients with pre-existing keratopathy, thus excluding numbers of patients with uveitis because coexisting uveitis and keratopathy in viral infections are not uncommon [30,31]. Among systemic conditions, cardiovascular as well as pulmonary disorders are the major disorders associated with a higher probability of keratopathy, but the exact mechanism needs further validation.

There are several limitations to the current study. First, the observational and retrospective nature of the study design may diminish the standardization of the patient population, even after multivariate analysis. In addition, we used claim data rather than real medical documents, and some important information, such as the laterality and severity of keratopathy and the postoperative condition of CRS after the FESS procedure, could not be accessed. Moreover, there are different types of keratopathy (e.g., infectious keratitis or corneal edema), and we failed to analyze the effect of surgery-indicated CRS on each type of keratopathy due to extremely small numbers for certain types of keratopathy.

## 5. Conclusions

In conclusion, surgery-indicated CRS that requires FESS to relieve symptoms is a potential risk factor for keratopathy after adjusting for multiple potential risk factors. Furthermore, the risk of developing keratopathy in patients with surgery-indicated CRS is correlated to a longer disease period of CRS. Further study to investigate whether surgery-indicated CRS leads to a worse prognosis for keratopathy is advocated.

## Figures and Tables

**Table 1:** Baseline characteristics between the study and control groups.

Baseline Characteristics	Study, *n* = 6053	Control, *n* = 24,212	*p* Value
Age			1.0000
<40	2177 (35.97%)	8708 (35.97%)	
40–59	2643 (43.66%)	10,572 (43.66%)	
60–79	1168 (19.3%)	4672 (19.3%)	
≥80	65 (1.07%)	260 (1.07%)	
Sex			1.0000
Male	3812 (62.98%)	15,248 (62.98%)	
Female	2241 (37.02%)	8964 (37.02%)	
Co-morbidities			
Hypertension	1662 (27.46%)	5654 (23.35%)	<0.0001
Diabetes mellitus	823 (13.6%)	2838 (11.72%)	0.0047
Ischemic heart diseases	634 (10.47%)	1901 (7.85%)	<0.0001
Hyperlipidemia	1466 (24.22%)	4736 (19.56%)	<0.0001
Heart failure	223 (3.68%)	675 (2.79%)	0.0002
Peripheral vascular disease	165 (2.73%)	554 (2.29%)	0.0454
Cerebrovascular disease	432 (7.14%)	1408 (5.82%)	0.0001
Dementia	37 (0.61%)	167 (0.69%)	0.5045
Chronic pulmonary diseases	1701 (28.1%)	3920 (16.19%)	<0.0001
Rheumatic disease	154 (2.54%)	414 (1.71%)	<0.0001
Peptic ulcer disease	1839 (30.38%)	5352 (22.1%)	<0.0001
DED	401 (6.62%)	1024 (4.23%)	<0.0001
Uveitis	54 (0.89%)	179 (0.74%)	0.2237
Glaucoma	161 (2.66%)	386 (1.59%)	<0.0001

DED = dry eye disease.

**Table 2:** The incidence of keratopathy in the study and control groups.

Incidence	Study, *n* = 6053	Control, *n* = 24,212
Follow-up person months	310,709	1,258,744
New keratopathy events	231	695
Incidence rate * (95% CI)	74.35 (65.35–84.58)	55.21 (51.26–59.48)
Crude Relative risk (95% CI)	1.345 (1.159–1.561)	Reference

CI = confidence interval; * Incidence rate, per 100,000 person months.

**Table 3:** Multiple Cox proportional hazard regression for the estimation of adjusted hazard ratios on keratopathy.

Variable	aHR (95% CI)
Surgery-indicated CRS	1.208 (1.038–1.406)
Age (Reference: 40–59)	
<40	1.044 (0.885–1.231)
60–79	1.487 (1.249–1.77)
≥80	1.06 (0.535–2.099)
Sex (Reference: Female)	
Male	0.648 (0.568–0.739)
Co-morbidities	
Hypertension	1.235 (1.034–1.475)
Diabetes mellitus	0.946 (0.771–1.161)
Ischemic heart diseases	0.983 (0.783–1.235)
Hyperlipidemia	1.085 (0.906–1.300)
Heart failure	0.852 (0.595–1.219)
Peripheral vascular disease	1.297 (0.921–1.827)
Cerebrovascular disease	0.953 (0.732–1.241)
Dementia	0.874 (0.427–1.792)
Chronic pulmonary diseases	1.335 (1.142–1.561)
Rheumatic disease	1.261 (0.874–1.821)
Peptic ulcer disease	1.105 (0.946–1.292)
DED	2.085 (1.668–2.607)
Uveitis	1.414 (0.792–2.524)
Glaucoma	1.334 (0.93–1.914)

aHR = adjusted hazard ratio; CI = confidence interval; DED = dry eye disease.

**Table 4:** The sensitivity analysis for the adjusted hazard ratio stratified by sex and age groups.

Subgroups	Incidence Rate (95% CI) of Keratopathy		aHR (95% CI)
	Study	Control	
Sex subgroups			
Male	55.88 (46.39–67.3)	44.87 (40.47–49.76)	1.14 (0.919–1.415)
Female	107.09 (89.54–128.07)	73.39 (65.94–81.69)	1.287 (1.038–1.595)
*p* for interaction			0.5209
Age subgroups (at index date)			
<40	52.03 (40.56–66.74)	44.56 (38.97–50.94)	1.117 (0.838–1.488)
40–59	72.26 (59.34–87.99)	48.89 (43.42–55.04)	1.295 (1.024–1.637)
60–79	125.9 (98.91–160.26)	96.41 (84.02–110.63)	1.148 (0.862–1.527)
≥80	189.49 (71.12–504.88)	53.07 (22.09–127.49)	12.006 (1.004–143.496)
*p* for interaction			0.4097

aHR = adjusted hazard ratio; CI = confidence interval.
